# Understanding the Influence of Early-Life Stressors on Social Interaction, Telomere Length, and Hair Cortisol Concentration in Homeless Kittens

**DOI:** 10.3390/ani15030446

**Published:** 2025-02-06

**Authors:** Jennifer Vernick, Chelsea Martin, William Montelpare, Arthur E. Dunham, Karen L. Overall

**Affiliations:** 1 Department of Health Management, Atlantic Veterinary College, University of Prince Edward Island, Charlottetown, PE C1A 4P3, Canada; 2 Department of Microbiology and Pathology, Atlantic Veterinary College, University of Prince Edward Island, Charlottetown, PE C1A 4P3, Canada; ckmartin@upei.ca; 3 Department of Applied Human Sciences, Faculty of Science and Faculty of Nursing, University of Prince Edward Island, Charlottetown, PE C1A 4P3, Canada; wmontelpare@upei.ca; 4 Biology Department, University of Pennsylvania, Philadelphia, PA 19104, USA; adunham@sas.upenn.edu

**Keywords:** homeless kittens, hair cortisol concentration, relative telomere length, epigenetics, early-life stress, neurodevelopment

## Abstract

The early postnatal period is a critical neurodevelopmental stage characterized by rapid neural maturation and is adversely affected by early-life stressors. This study explored the behavioural, physiological, and epigenetic consequences of early-life stress in a population of homeless rescue kittens. This longitudinal study included 50 kittens rescued and placed into foster care by the Prince Edward Island Humane Society. They underwent behavioural testing at 8, 10, and 12 weeks of age. Hair cortisol concentration was measured at 8 weeks and served as a physiological marker of the previous 3 months’ cumulative stress response, which, for these kittens, included the late gestation period. A blood sample for relative telomere length measurement was taken at 10–12 weeks to estimate epigenetic changes as young kittens. Data were analyzed with respect to age and performance in all repeated measures tests, status as a stray or a surrender, and the presence of the dam in their foster homes. As expected, the performance of kittens in all tests changed over the 5 weeks of testing. Kittens separated from their mothers exhibited significantly higher hair cortisol concentrations (*p* = 0.02) and elongated relative telomere lengths (*p* = 0.04). No correlation was found between hair cortisol concentration and relative telomere lengths (*p* = 0.99). These results support the need for further study on the effects of epigenetics and early-life stress, both in kittens and across species.

## Simple Summary

Early-life stress, such as inadequate and unpredictable nutrition, disease, poor housing, and insecurity about safety, affects aspects of brain development and later behaviours across species. Few data exist on the effects of such maternal and early-life stress in kittens, which is remarkable, given the number of stray cats and homeless kittens who face such stressors. This study explored the effects of early-life stress on some physiological markers and also on a range of behaviours in a population of homeless rescue kittens under the care of the Prince Edward Island Humane Society. The results of this study showed significant differences in the behavioural responses to humans in testing performed across the first 3 months of life and physiology of kittens associated with their early-life experience, such as early maternal separation or life as a stray. The behaviours of these rescue kittens differed from those of well cared for laboratory-raised kittens, further highlighting the potential effects of early-life stress. The findings of this study suggest that interactions between early-life stress, physiological responses, behaviour, and epigenetic changes in domestic kittens are complex and warrant in-depth evaluation.

## 1. Introduction

The early postnatal period is a critical phase characterized by rapid neural maturation and heightened susceptibility to environmental and social influences. This time frame is viewed as a “sensitive period” for neuronal and behavioural development [1,2] across species, including in domestic cats [3,4,5]. During this critical developmental window, the brain demonstrates exceptional sensitivity to environmental stressors, including stressors from the social, physical, and nutritional environments. These sensitive periods occur while offspring heavily rely on maternal care for their survival and optimal neurobiological development [4,5]. The health of the mother and whether she or her progenitors have encountered profound deprivation, neglect, abuse, and other stressors pre-, peri-, and postnatally have become a focus of epigenetic research for numerous species [6,7,8,9].

The influence of early-life stress on brain function, immune function, and later individual behavioural responses and behavioural pathologies has been amply demonstrated [10,11,12,13,14]. Early-life stress has been shown to result in deficits in dendritic branching and synaptic connections in the hippocampus, contributing to lifelong memory deficits [15]. Cognitive, emotional, and social functions [7,8,16,17,18] are impaired in individuals with transgenerational trauma [8,19], in utero nutritional deprivation [20], and profound in utero and early-life stressors [21,22]. Early-life stress induces lasting modifications in neural circuits and brain regions, particularly those closely associated with the stress-response system [7,8,23,24,25,26,27]. Conversely, enhanced maternal care during early life, characterized by increased sensory input, is associated with long-term reductions in neuroendocrine and behavioural responses to stress, enhancing resilience against depression, and augmented cognitive function [28].

In kittens, early-life stress can lead to various behavioural and developmental abnormalities, including learning deficits, abnormal fear responses, and increased aggression [29]. Early weaning and/or inadequate in utero nutrition may contribute to epigenetic effects. Early nutritional stress has been associated with early predatory behaviour in kittens [30,31], alterations in play behaviour [32,33,34], and enhanced reactivity, fear, aggression, and incoordination [34,35,36]. A decrease in food intake in the dam of 50% at gestation produces kittens with delayed postural corrections, walking, running, climbing, and play [33]. These kittens have been noted to have delayed exploratory behaviours, poor learning ability, increased reactivity, abnormal fear and aggression, and decreased responsiveness to normal environments. Queens fed 50% of their ad libitum intake during the second half of gestation and through the first 42 days of the kittens’ lives produce kittens with abnormal play behaviour. These kittens have more accidents in play, have more aggression in social play, and have abnormal brainstem, cerebellar and cerebral growth [32]. Low-protein diets during late gestation and during lactation produce kittens with delayed development that are less coordinated in their movements, engage in fewer social interactions with their mothers, and have less attachment to their mothers [37,38]. These responses are concordant with those reported for in utero and very early-life stressors reported in other species [20,21,22].

There are few data on effects of early environments on performance parameters in kittens, and none that use exactly the same methodology. Kittens handled regularly for the first 45 days (6.4 weeks) of life approach unfamiliar objects more rapidly and spend more time with them at 4–7 months than unhandled kittens [39]. Kittens handled by people for 15 min per day from birth through 12–14 weeks spent more time exploring the person and giving head rubs at 14 weeks [40,41]. Sensitive periods in kittens begin early, so handling them between 2 and 9 weeks increases comfort with humans and decreases fear, and kittens handled for augmented lengths of time through 14 weeks can compensate for lack of early handling [3]. Early exposure to humans enables cats to have greater behavioural flexibility in these situations and to be less fearful [39,40,41].

The only data to investigate age-specific effects of types of handling and approaches to humans are those of Karsh [3,40,41,42] who studied well-cared-for laboratory cats. Martinez-Byer et al. [43] investigated handling/struggling, food defence, and separation/confinement responses in 62 9-week-old rescue kittens from varied rearing environments. Marchei et al. [44,45] studied the responses of Norwegian forest cats and Oriental/Siamese/Abyssinian kittens aged 4–10 weeks in their home environments in an open field test and in response to threat when tested in the open field (see summary of results in Table 1).

Understanding the effects of early-life stress on kitten behaviour and potential later-life behavioural problems is crucial, not only as models for effects of neurodevelopment, stress, and deprivation on human behaviour, but also for feline welfare, as behaviour problems are frequently cited as the primary reasons for cats being surrendered or abandoned [48].

Across species, early maternal care has been shown to affect resilience or vulnerability to cognitive and emotional deficits, many of which are modulated through the activation of the hypothalamic adrenal axis (HPAA) [16,23,49]. Augmented maternal care has been associated with the suppression of corticotropin-releasing hormone (CRH) expression in the hypothalamus preceding glucocorticoid receptor (GR) expression in hippocampus, which allows responses to stressors to be aborted in a timely manner as part of an adaptive return to a homeostatic state [23]. Neonatal rats show such suppression and subsequent functional regression of the HPA-axis between 4 and 14 days postnatally, resulting in baseline levels of plasma glucocorticoids that are far lower than normal and rise only minimally in response to stressors that would normally induce a robust rise in glucocorticoid levels in an adult [50]. This restricted postnatal period during the first 2 weeks of life in rodents [51] has been referred to as the stress-hyporesponsive period (SHRP). The SHRP is thought to be an adaptation to maintain stable and low levels of corticosterone during early development in order to promote normal central nervous system (CNS) development across species [49,52,53]. A prolonged SHRP has been associated with maternal presence and care [54] and may delay glucocorticoid elevation in infants, decreasing any pronounced stress response that could affect later behaviours. This pattern has been demonstrated for the SHRP in dogs, which have the same gestation period as cats (mean = 63 days). Pronounced maternal care lengthens the innate SHRP from post-natal week 4 to postnatal week 5 in dogs [52]. There are no reported investigations of a SHRP in kittens.

Deprived/inadequate care has been shown to lead to heightened CRH expression, decreased GR expression in the hippocampus, increased serum cortisol, and a chronic stressor response, including high levels of glucocorticoids, impaired cognition, and heightened emotional reactivity [24,55,56,57]. In such situations, the chronicity of excessive glucocorticoid exposure may lead to low levels of circulating corticosteroids (e.g., a blunted cortisol response) due to cortical suppression [27,58,59,60]. Early elevated cortisol levels may induce persistent, rather than transient, changes in HPA-axis function [10,61]. Latently expressed behavioural alterations including deficits in sensorimotor gating [62], long-term depression-like behavioural traits [63], anxiety-like behaviours [64], increased neophobia [65], and impairments in aversive memory [57] may be sequelae.

Homeless and/or orphaned kittens may be at risk of early (transgenerational, in utero, peri- and postnatal) aversive and/or deprivational social, physical, and nutritional environments that increase the risk of cortisol-mediated neurodevelopmental insults. Such risks may manifest as “problem” behaviours in adult cats and extreme or pathological behaviours in kittens as they mature [66].

Cortisol can be measured in blood, plasma, urine, saliva, feces, and hair [67], and each method has different optimal applications and limitations [68,69]. Hair cortisol concentration is a minimally invasive, retrospective measure of cumulative HPA activity over the preceding 3 months and is increasingly viewed as an emergent biomarker of HPA activation and response to stressors [70,71,72]. Hair cortisol measurements have been reported across wild felid species [73,74] and are considered valid and reliable measures in domestic cats [75,76].

Epigenetic effects refer to changes in gene expression or cellular traits that arise from modifications in phenotype or gene expression [77]. Examples of epigenetic measures include DNA methylation patterns, histone modification, and the regulation of gene expression by non-coding RNAs [78,79,80,81]. Early and prolonged extensive maternal care has been shown to decrease DNA methylation [54].

Telomere length is a surrogate marker for epigenetic effects occurring in utero and in early life [20]. Telomeres are repetitive nucleotide sequences at the end of chromosomes that protect them from DNA repair and degradation and shorten with replication of somatic cells (i.e., ageing) [82]. In humans, maternal stress during pregnancy is associated with shorter telomeres in the offspring, which, in turn, is correlated with increased incidence of psychiatric disorders [21]. Exposure to adverse childhood experiences has been associated with adverse psychological effects, the dysregulation of neurochemical systems, including the HPA axis, and shortened telomeres [83,84]. The telomere effect is pronounced if physical neglect was involved, as is the case with many homeless kittens [85]. Kittens may also be exposed to environmental toxins, such as metals and organic compounds, which also shorten telomeres [86]. Telomeres are shorter in older cats and have a pattern of expression similar to that found in humans, but the average telomere length in cats is 5–10 times longer than in humans and rates of telomere shortening is much higher [87], suggesting that telomere measures may be informative.

A recent study found no significant difference in telomere length between 42 orphaned and 10 mother-reared kittens from animal rescue groups or shelters when telomeres were measured at 8 weeks [88], although the range of telomere lengths were more variable for orphaned than for mother-reared kittens. Regardless of maternal presence, the kittens in that study were all reared in care from their first week. Kittens of homeless mothers that were their sole caretakers for at least the first few weeks, as was the case with several of the PEIHS kittens, may have experienced very different stressors than those raised in care from a week of age onward. Kittens reared without human intervention by homeless mothers may be exposed to inadequate pre- and peri-natal diets, variable shelter environments, threats, and toxins, and severity of these may vary across the breeding season.

Cats have emerged as promising models for studying human neuropsychiatric conditions due to their natural display of analogous mental health challenges [89,90]. The development of temperament and behaviour in cats, like other animals, is influenced by both genetic and environmental factors [36,41,91]. A significant gap persists in our understanding of the long-lasting stress effects on epigenetics and behaviour that manifest during the early stages of the life of kittens and other domestic species.

Accordingly, this study sought to investigate the behavioural, physiological (hair cortisol concentration), and epigenetic consequences (relative telomere length) of exposure to early-life stressors in cats by testing 50 kittens rescued and placed into foster care by the Prince Edward Island Humane Society (PEIHS). All kittens were born to homeless mothers, so we hypothesized that all kittens were subject to a range of in utero and peri-natal stressors. We hypothesized that this range would be reflected in the responses to a series of interactive and non-interactive behavioural tests and that the outcomes of the interactive tests discussed here (feather test, approach to an unfamiliar human test, and holding by an unfamiliar human test) would correlate with demographic variables (presence or absence of dam in shelter and in the foster home, presence of cats or dogs in the foster home, number of days in foster care, and source (surrender or ‘stray’) and with physiological measures of early stressors (hair cortisol concentration (HCC) and relative telomere length (RTL)). We also hypothesized that RTL, an epigenetic marker of in utero stressor exposure and HCC, a marker of the cumulative effects of stressors over the past 3 months on cortisol response, would correlate, that kittens with high HCC levels (indicative of higher stress response) would have shorter RTLs, and that those with lower HCC would have longer RTLs. Finally, we hypothesized that, with increasing numbers of test periods and ageing past the late play and human social exposure periods (Table 1 [3,40,41]), kittens, regardless of their performance at 8 weeks, would have a shorter latency to approach the feather toy and play with it longest at 12 weeks and also have a shorter latency to humans and allow themselves to be held longer.

All kittens were tested at 8, 10, and 12 weeks across a series of tests designed to evaluate environmental exploration, interactive engagement, and social responses to novel humans. This paper focuses on interactions with a moving object and unfamiliar humans. A subsequent paper will focus on the effects of age and rearing on environmental interactions. Our research advances the understanding of early-life stress on behavioural ontogeny and epigenetics and has practical implications for the management and welfare of feral cat populations.

## 2. Materials and Methods

This study was conducted over 4 months from the end of June through mid-September 2022, peak kitten season on Prince Edward Island (PEI). The PEIHS has a long-standing program where homeless kittens are collected by the PEIHS after being reported, surrendered, or brought to the PEIHS by good Samaritans. Attempts are made to retrieve the kittens with their dam, and all cats are registered at the PEIHS, and when they are healthy, they are placed into foster care as soon as possible. Prince Edward Island (PEI) has a large network of foster-carers who have repeatedly participated in the program. When the program fully restarted in 2022 after being paused in 2020 due to COVID, we worked with the PEIHS to enrol 50 kittens into our study. All procedures were conducted in accordance with the guidelines established by the Canadian Council on Animal Care and were approved by the Animal Care and Use Committee at the University of Prince Edward Island (UPEI) (ACC Approval Number 22-004). All foster guardians gave informed consent prior to participating in the study and were instructed that they could withdraw kittens under their care from the study at any time. At the completion of the study, all foster guardians received a gift card valued at CAD 50.00 per litter for their participation.

### 2.1. Subjects

The participants were 50 kittens of homeless (and possibly feral) mothers that had been rescued at a range of ages (0–52 days). Seventeen were born in either foster care or at the PEIHS (Charlottetown, PE, Canada) and subsequently placed into foster care by the PEIHS. Of the kittens recruited to participate in this study, 24 were female and 26 were male (48% and 52%, respectively). A power analysis based on holding (median = 14.3 s; mean = 28.81; SD = 27.28 s) and approach (median = 5.9 s; mean = 13.83 s; SD = 23.84 s) times for a partial initial sample of 31 kittens showed that the power of the test ranged from 0.95 to 1.00, respectively.

In addition to the 50 kittens discussed above, blood samples were collected from an additional 41 later-season kittens; 32 samples were adequate to measure RTL. These kittens presented at the HS at a range of ages (0–162 days; mean = 76 days; SD = 19.1 days) and consisted of 20 females and 21 males (49% and 51%, respectively). These kittens did not participate in any behavioural testing and no questionnaires were completed for them.

As part of the PEIHS’s operational procedures, all kittens were neutered at approximately 10 weeks of age; thus, all participating kittens were neutered during the course of this study. However, with the exception of one male kitten that was neutered two days before testing at 10 weeks of age, no kittens were tested within 4 days of surgery.

It was the PEIHS’s standard operating procedure to keep kittens with their mothers, if their mothers were rescued with them and could adequately care for them. When this was not the case, the PEIHS attempted to cross-foster singletons into other intact litters, and, on occasion, larger litters were divided and fostered in different homes. Dams were present with the foster kittens for at least part of the duration of this study for 21 (42%) of the kittens.

### 2.2. Experimental Setting

All testing took place between 0800 and 1500 h on the campus of the Atlantic Veterinary College (AVC) at the University of Prince Edward Island (UPEI; Charlottetown, PE, Canada). Each kitten was tested at approximately 8 weeks (7–9 weeks; average age in days = 60.72; SD = 4.33 days), 10 weeks (9–11 weeks; average age in days = 75.0; SD = 4.93 days), and 12 weeks (11–13 weeks; average age in days = 89.0; SD = 4.24 days).

The testing and holding rooms, where all procedures took place, were adjacent to one another, climate controlled, and located in a restricted-access area. The holding room was maintained at 21.35 °C (±1.62) with 62.79% (±6.32) humidity. Background noise in the holding room was consistent at approximately 49 dBs, and lighting was maintained at approximately 142 lux (preset default) for the study duration. Contained within the holding room were four large metal cat cages (Veterinov^TM^ Veterinary Cat Condo; Veterinov, Sherbrooke, Quebec, Canada).

Litters of kittens were transported to the facility in their own large plastic travel kennels provided by the PEIHS when they went to foster. All kittens were familiar with the kennels. Kittens were transported by car by the fosters for no more than 30 min to the facility. This type of repeated travel is one of the recommendations made to expose kittens to novel environments and situations under secure and protected situations [12].

When each litter of kittens arrived for testing, they were transported into the building inside their large plastic travel kennels, where they were allowed to remain until transferred to the holding cage within 10 min. Once inside the holding room, they were moved one at a time from the travel kennels and housed with their littermates in one of the cages where they had free access to a litter box and water. Kittens drank the water, and some used the litter box upon arrival, which are normal behaviours for kittens this age. A clean, fluffy blanket was placed in the main area of each cage. Each cage (71.12 × 73.66 × 71.12 cm) was equipped with an elevated shelf (40.6 cm from the floor) and an attached but separate area (25.40 × 73.66 × 71.12 cm) which contained a litter box. On occasions where kittens arrived more than one hour prior to testing, they were given ad libitum access to a high-quality kitten kibble (ACANA^®^ First Feast Kitten Cat Food) in multiple dishes and monitored to ensure that no one kitten controlled access to the food. In such cases, food was removed from the holding kennels 30 min prior to the commencement of testing for that litter. In all cases, once all members of a litter had been tested, food was placed in the holding kennel, and ad libitum access to the food, water, and litter was given until litters were returned to their foster guardian. The food was almost always consumed, and the water dishes and litter boxes were used. These are normal behaviours for young kittens, suggesting that the testing situation was not perceived by them as adverse or overly stressful.

Prior to testing, each kitten was gently individually removed from the cage and transported to the adjacent testing room in a soft-sided travel carrier (26 × 30 × 48 cm). The environmental conditions of the test room were monitored daily for the duration of the study and room parameters were as follows: temperature: 22.5 °C (±2.69), humidity: 59.79% (±11.23), background noise: 47.95 dBs (±1.38), lighting: 142 lux. While still in the travel carrier, each kitten was placed on the floor and left undisturbed (the location of the placement of the carrier was kept constant throughout) for a 5 min acclimation period. After the 5 min had elapsed, the kitten was gently removed from the travel carrier, photographed for identification purposes, and then assessed using three discrete behavioural tests (i.e., open field with an interactive object, approach to a novel human, and holding by a novel human; tests described in detail below). Between each behavioural test, the kitten was removed from the test arena and placed in the travel carrier (i.e., in the same location as above) for a 5 min inter-test interval before beginning the next test paradigm.

### 2.3. Behavioural Testing

**Figure 1:**
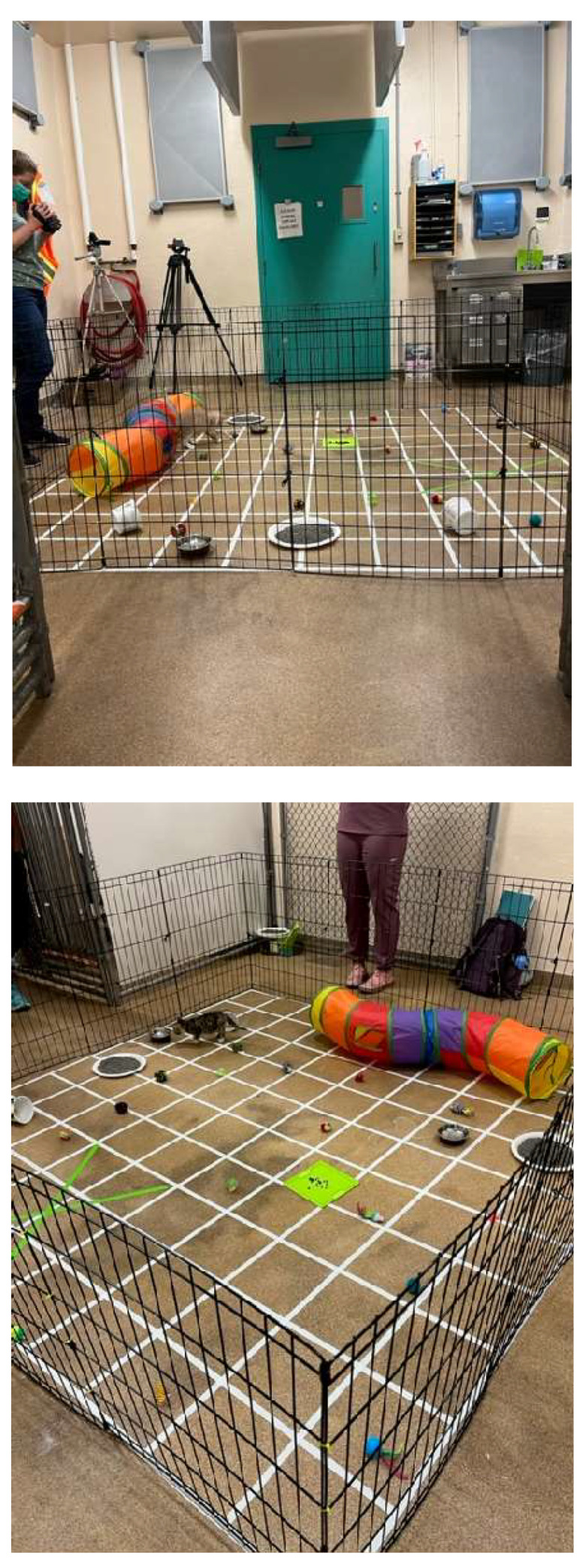
Two views of the open field testing set up taken from opposite sides of the room. The interactive object/feather test occurs in the region marked with an X.

All behavioural tests took place in the same test arena (2.44 × 2.44 m, divided into n = 100 0.24 m grids, with the walls of the arena defined by 92 cm high “X-pen” panels; see photos Figure 1). Behavioural assessments were conducted in the same sequence for all kittens over each of the three sessions (i.e., 8, 10, and 12 weeks of age). The arena and all toys and bowls used during testing were cleaned/disinfected between each litter tested. The floor and the toys were cleaned/disinfected with Prevail^®^, and food and water bowls were thoroughly cleaned with commercial dish detergent. All behavioural testing was video-recorded for subsequent scoring.

#### 2.3.1. The Interactive Object/Feather Test (1 min)

**Figure 2:**
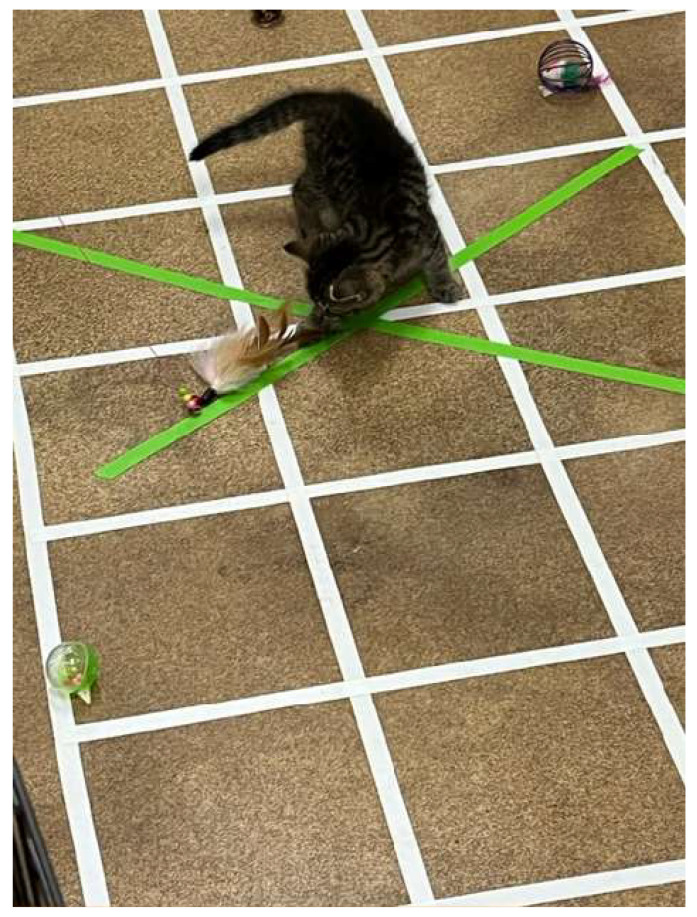
Kitten playing with the feather toy. The feather is on a wand, the experimenter is outside the X-pen, and the feather is slowly moved from the most distal end of the arm of the X, proximally, for first one arm, then the other. This action is repeated for the full minute.

The feather test was the last part of the 5 min open field and interactive object test. After 4 min of exploration of the field and the items within it, the interactive object test was introduced. This portion of the test was conducted by an experimenter located outside the arena. The interactive object here was a long wand toy with feathers and bells. The experimenter, holding the wand toy, inserted the feathered end through the arena wall and moved it in an ‘X’ pattern along a pre-marked course within the arena space (see Figure 2). The experimenter did not directly interact with the kitten during this test. This test was used to assess interaction with a predictably moving object since object play and willingness to explore moving objects—separately from interest in and interaction with humans—may be impaired if maternal care is insufficient [26,32,34,66,91]. This portion of the test lasted for 1 min. Upon completion of the test, the kitten was gently collected by a researcher and placed back into the travel carrier for a 5 min interval (i.e., before commencing the approach to a novel human test).

#### 2.3.2. Approach to a Novel Human Test (2 min)

**Figure 3:**
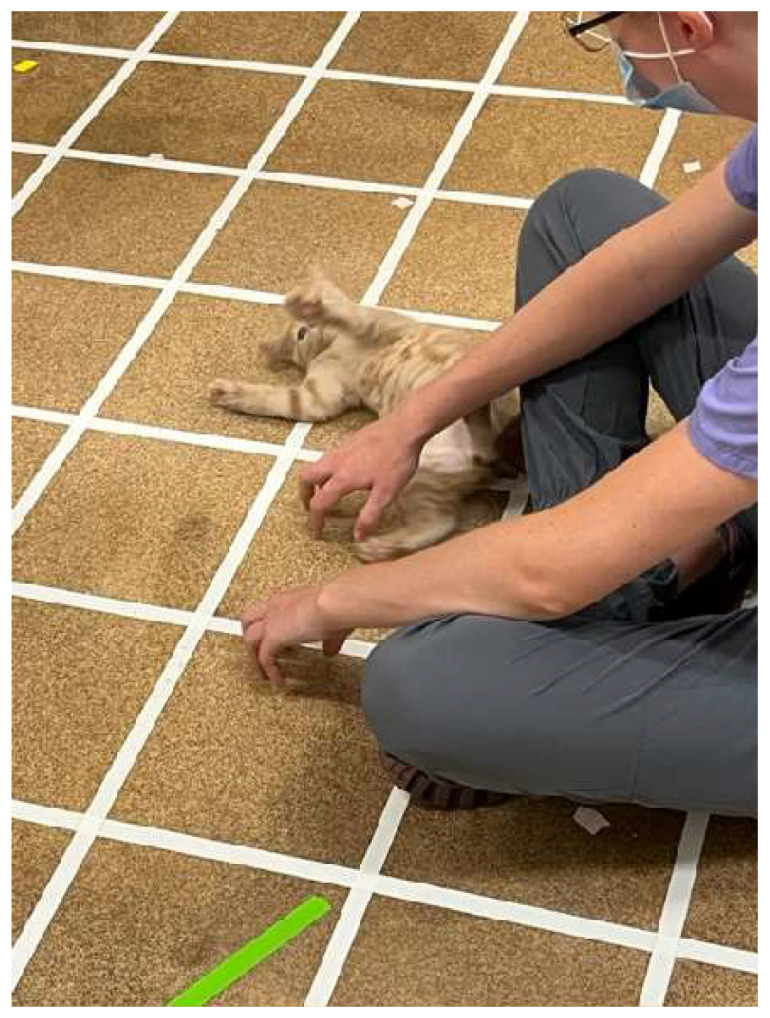
Kitten during the finger tapping part of the approach test.

The approach test was adapted from Karsh and Turner [3] because these are some of the only published, detailed, experimental data across ages of kittens with respect to human interactions. For this test, an experimenter was seated at a pre-marked location within the arena. The kitten was removed from the travel carrier and placed into the arena at a pre-marked location approximately 120 cm away from the experimenter. Latency (s) to approach the novel human was recorded as the time from when all four feet were on the ground to when the kitten made physical contact with the human. For the first minute, the experimenter sat quietly without making any attempts to interact with the kitten. After 60 s had elapsed, the experimenter gently tapped on the floor for an additional 60 s, in an attempt to engage the kitten (Figure 3). Once testing was complete, the kitten was collected and placed back into the travel carrier.

#### 2.3.3. Holding by a Novel Human Test (2 min)

**Figure 4:**
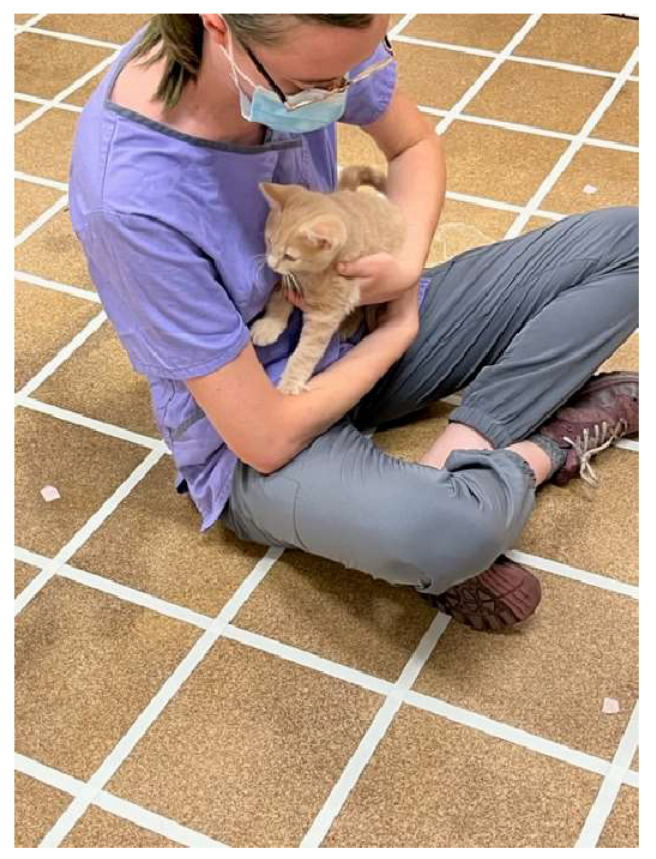
Kitten being gently supported for holding in the lap of the experimenter.

The holding test was adapted from Karsh [41] and Karsh and Turner [3] because these are some of the only published, detailed, experimental data across ages of kittens with respect to human interactions. The total duration of this assessment was 2 min. Here, an experimenter was seated on the ground within the arena, approximately 25 cm from and facing the arena wall. The kitten was removed from the travel carrier and gently handed to the experimenter to be placed in their lap. The experimenter began to softly stroke the kitten from head to tail without any attempt to forcefully restrain the kitten while picking it up fully supported (Figure 4). If/when the kitten left the experimenter, the experimenter would wait until the kitten returned to within “easy reach” proximity (about 12 cm) to pick him/her up for another holding attempt, again ensuring that the kitten was not forcibly restrained in any way. A maximum of 3 holding attempts were permitted for each kitten during a test session. Holding duration for any attempt was measured as the time (s) from when the kitten was settled in the experimenter’s lap, held by their arms, until all four feet touched the ground. After the 2 min had elapsed, the kitten was placed back into the travel carrier, transported to the holding room, and was returned to the kennel with the rest of his/her foster group.

### 2.4. Physiological Assays

#### 2.4.1. Hair Cortisol Concentration (HCC)

Hair cortisol concentration was used as a surrogate for response to stressors by providing a 3-month cumulative measure of pre/peri/post-natal stress responses (last month in utero through 8 weeks). Hair samples for hair cortisol concentration measurement were collected from all kittens after the completion of the behavioural testing at their first visit (8 weeks). A 3 × 3 cm patch of fur was shaved from the right lateral shoulder region using commercially available clippers (WAHL, Mini Arco, Sterling, IL, USA). Kittens were wrapped in a soft blanket (‘burritoed’), and before and after shaving, kittens were offered tiny pieces of dried whitefish, which they usually accepted. They were placed in their cages and offered food after shaving. The hair from each kitten was collected, wrapped in aluminum foil labelled as to cat and date, and stored in a dark drawer. All samples were kept in a climate-controlled room until they were shipped to the Drug Safety Laboratory, Robarts Research Institute, Western University, ON, Canada, with information about the kitten’s ID number, age, hair colour, and date of sample collection,

#### 2.4.2. Relative Telomere Length (RTL) Assessment

Telomere length was used as a surrogate for kitten in utero and perinatal maternal stress. Relative telomere length (RTL) from whole blood samples was used to estimate the epigenetic effects of pre/peri-natal stress on any relevant test performance variables and behaviours. RTL was measured using quantitative polymerase chain reaction (qPCR), using methods adapted from published literature [88,92,93,94]. Telomere sequence (TTAGGG)n is highly conserved among animals [86]. The primer sequences used in this study (TeloS: CGGTTTGTTTGGGTTTGGGTTTGGGTTTGGGTTTGGGTT; TeloAS: GGCTTGCCTTACCCTTACCCTTACCCTTACCCTTACCCT) have been used to evaluate RTL in a variety of species [93,95,96]. RTL was calculated as telomere expression relative to expression of a single copy reference gene (hypoxanthine phosphoribosyltransferase 1 (HPRT1). Pooled DNA from 10 kittens served as a reference sample for all reactions. All blood samples were obtained from when kittens were between 10 and 12 weeks of age. If the kittens were due to be neutered, the veterinarian responsible for the neutering obtained the 0.5 mL sample while the kitten was anesthetized. If the kitten had already been neutered but blood had not been collected at that time, blood was collected at the last visit (12 weeks) by the authors from the medial saphenous vein, using a 23-gauge needle, 20 min after the application of topical 2.5% lidocaine/2.5% prilocaine cream. Pieces of freeze-dried whitefish (PureBites Whitefish, Sacramento, CA, USA) were sprinkled on the table to occupy the kitten who was gently positioned on a blanket while being stroked and fed. Blood was stored in lithium heparin tubes and refrigerated prior to DNA extraction. There were 49 RTL measurements from kittens in the study; one kitten in the study did not have a sufficient sample. In addition to the study kittens, we were able to perform RTL analysis on blood samples taken from 32 kittens brought to the PEIHS later in the season, for a total of 81 telomere measures.

### 2.5. Data Analyses

Data analyses were conducted using JAMOVI (v. 2.3.26), Social Science Statistics (https://www.socscistatistics.com/, accessed on 30 December 2024) and R+. Latency (seconds) to approach a novel human was recorded for each kitten tested in the approach test. If kittens did not approach, then we considered them to not have completed the test. The data for HCC, telomeres, and other potential social factors were separately evaluated for kittens who approached the novel human and those that did not approach the novel human at 8, 10, and 12 weeks. Calculations of time to approach humans included only kittens who made physical contact with the human, no matter how brief. In the holding by a novel human test, the longest hold time (seconds) of up to 3 holding trials over the 2 min test period was used as the holding time metric.

Behavioural factors analyzed included latency to interact with the feather toy, time engaged with feather toy, latency to approach humans, and holding time by a novel human.

Demographic factors analyzed included: presence or absence of dam at presentation to the human society and at the time of 8-week testing, interactions with dogs and/or cats in the foster home, intake status (i.e., “stray” vs. “surrender”), and number of days in foster care before the 8-week testing. The physiological measures consisted of HCC and RTL.

For behavioural and physiological assessments, unless otherwise stated, data for all variables were analyzed through repeated measures of ANOVAs or Friedman tests. Additionally, paired comparisons were analyzed with *t*-tests. Pearson correlations were used to compare HCC and RTL. Effects of dam and interactions with cats or dogs in the foster household were considered.

## 3. Results

### 3.1. Latency to Approach Feather Toy

Kittens had 60 s to approach and interact with the feather toy. Twenty-three (23/50) kittens did not approach the feather toy at 8, 10, and/or 12 weeks. Six kittens did not approach the feather toy at only 1 age, 13 kittens did not approach at 2 ages, and 4 kittens did not approach at any age.

When kittens that did not approach the feather toy at any age were removed from the analysis, the latency to approach the feather toy significantly decreased with age (repeated measures ANOVA: F (2, 78) =4.12, *p* = 0.02). One of these kittens also did not approach a human. When that kitten was removed from the analysis, the results were unchanged (repeated measures ANOVA: F (2, 77) =3.98, *p* = 0.025) (Table 2).

When comparing kittens who interacted with dogs in foster (N = 9) and those who did not (N = 19), there was no difference in latency to approach the feather toy, when kittens who did not approach the toy were removed. There was no difference in latency to approach the feather toy if the kittens interacted with pet cats (N = 17) in foster when compared with those who did not (N = 11), when kittens who did not approach the toy were removed. Interacting with dogs or cats had no effect on whether a kitten approached the toy when Z scores were compared. Despite this finding, kittens who interacted with dogs did not shorten their approach latencies across increasing weeks, but those who *did not* interact with dogs did (repeated measures ANOVA: F (2, 28) =5.96; *p* = 0.006).

### 3.2. Total Time Engaged with Feather Toy

Once kittens that did not approach the feather toy were removed from the analysis, a significant effect was found for age on the amount of time spent playing with the toy (repeated measures ANOVA: F (2, 78) = 17.93, *p* < 0.00001). When the one kitten who also did not approach a human was removed from the analysis, the results were unchanged (repeated measures ANOVA: F (2, 75) = 15.77, *p* < 0.00001) (Table 2). Three kittens played for the entire 60 s of the test at 8 weeks, 6 kittens did so at 10 weeks, and 8 did so at 12 weeks. One kitten played for the full 60 s across all weeks.

Separate *t*-tests were used to compare latency to engage with the feather toy and time spent playing with it by age for kittens with and without their dam present in their foster homes. At 10 weeks, kittens with the dam present were significantly faster at approaching the feather toy (t(25) = −2.16, *p* = 0.02). No other comparisons were significant (Table 3). There was no effect of presence of the dam on whether the kitten approached the feather toy.

When comparing kittens who interacted with dogs in foster (N = 9) and those who did not (N = 19), there was no difference in time spent playing with the feather toy, when kittens who did not approach the toy were removed. There was no effect of interacting with a dog on whether the kitten approached the feather toy. There was no difference in time spent playing with the feather toy if the kittens interacted with pet cats (N = 17) in foster when compared with those who did not (N = 11), when kittens who did not approach the toy were removed. Despite this finding, kittens who interacted with dogs did not play longer with the feather toy across increasing weeks, but those who *did not* interact with dogs did play longer (repeated measures ANOVA: F (2, 28) = 22.05. The *p* < 0.00001).

### 3.3. Approach to a Novel Human Test and Holding by a Novel Human Test

A total of 11 kittens did not approach the experimenter at least once across the three ages tested; 7 of these 11 kittens did not approach over multiple ages. At 8 weeks, 9 kittens did not approach; 3 of these 9 kittens did not approach across all ages tested.

Five kittens had extreme holding times (i.e., >120 s) because they burrowed into the experimenter’s lap (and were not actually ‘held’ in the sense of being in the experimenter’s arms). Because of this, holding time data were analyzed in four ways.

Holding time data were analyzed for *all* 50 kittens across all three ages, including the 5 kittens who had holding times of >120 s since we could not be sure whether these kittens were actively participating or simply hiding deep in dark laps, especially given the lack of willingness to approach at one or more time period for 4/5 of these kittens. Holding time data were analyzed *only* for kittens who approached within 120 s across all ages (N = 39). Holding time data were separately analyzed for the kittens that did not approach within 120 s for one or more time period (N = 11). Finally, holding time data were analyzed after removing *any* kitten with a holding time of > 120 s for 1 or more time period (N = 45) (see Table 2 for summary).

#### 3.3.1. Approach to Novel Human Test

**Figure 5:**
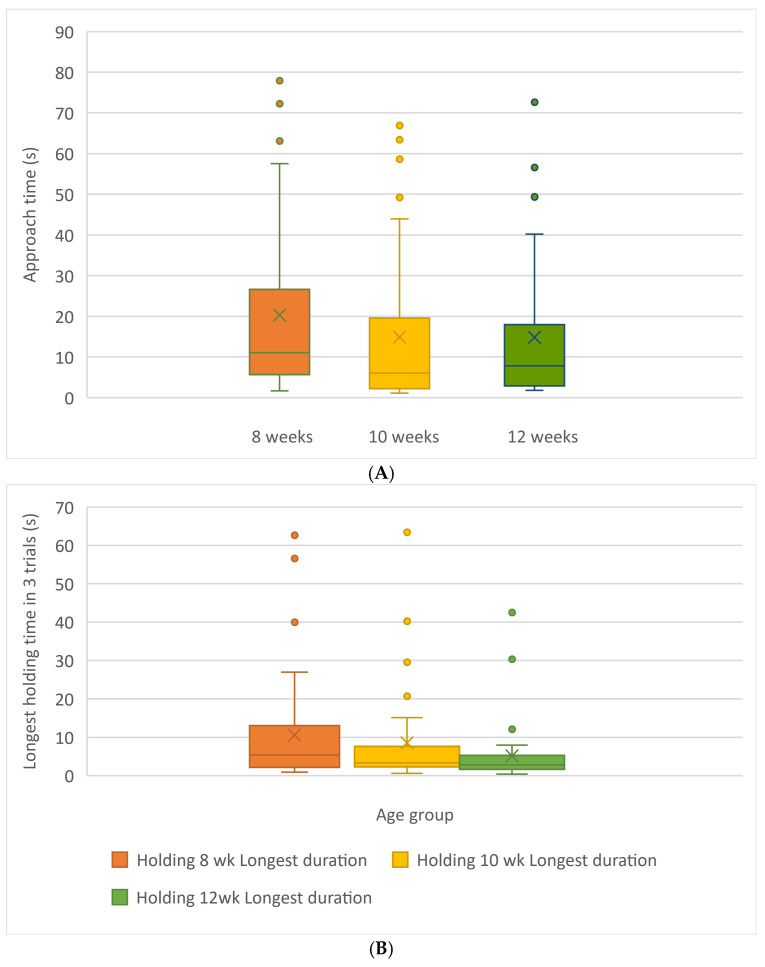
Distribution of approach (**A**) and holding (**B**) times for kittens. Kittens that did not approach were considered to not have completed the test and were excluded from the approach analysis (N = 39 approaching kittens across all ages). Kittens with extreme holding times (>120 s) in any interval, were considered to not have completed the test and were excluded from the holding time analysis (N = 45 kittens completing the holding test across all ages). See text for explanation. The box and whisker plots show the means (x), the medians (lines), the box contains 50% of the scores, and the values for 75% of index scores (whiskers) for each group. See text for SD/SE and statistical interpretation.

Using the data for kittens who approached, there was a significant difference in approach time with respect to kittens at 8 weeks compared to 10 and 12 weeks. Kittens approached a novel human more quickly at 10 and 12 weeks (Friedman test, χ^2^ _r_ = 7.54 (2,39); *p* = 0.02) than they did at 8 weeks, but there was no difference in approach time between 10 and 12 weeks (Table 2 and Figure 5A).

Interestingly, the majority of kittens who did not approach at any of the three test periods, also did not interact with the feather toy during that test period (Z score for 2 population proportions, Z = 3.33; *p* = 0.001). When comparing the frequency of not interacting with the feather toy for those kittens who did not approach, compared with those kittens that did approach, kittens that did not approach disproportionately did not interact with the feather toy (Chi square test; χ^2^ =24.13; *p* < 0.00001).

Of the 11 kittens that did not approach humans, only 1 approached the feather toy across all ages. Of the 23 kittens that did approach the feather toy at any age, 12 approached humans at all ages, while 11 did not approach humans for at least one age.

There was no effect of interacting or not, with either a pet cat or dog in foster care, on latency to approach a novel human.

With respect to whether the dam was present when the kittens came to the PEIHS (N = 27) or in the foster home throughout testing (N = 21), there was no effect of presence of a dam on whether the kitten approached a novel human (all NS Chi square tests). For the kittens that approached novel humans, there was no effect for any test period of the presence of the dam in the shelter or foster on the time it took the kitten to approach (all NS Kruskal–Wallis tests).

#### 3.3.2. Holding by a Novel Human Test

If all kittens, including those that did not approach the experimenter, are included in the analysis, the longest holding attempt significantly differed at each of the three ages tested and *decreased* with age (Friedman test *X^*2*^ _r_* = 13.81(2,50); *p* = 0.001) (Table 2).

If kittens that did not approach were removed from the holding time data across all weeks (N = 11), the longest holding attempt significantly differed at each of the three ages tested and *decreased* with age (Friedman test, *X^*2*^ _r_* = 7.54 (2,39); *p* = 0.02) (Table 2).

If only kittens that did not approach were analyzed for holding times, the holding times decreased significantly with each age (Friedman test *X^*2*^ _r_* = 7.68 (2,11); *p* = 0.02). These holding times were long because the kittens with holding times > 120 s were not removed and 4/5 of these kittens also did not approach (Table 2).

**Figure 6:**
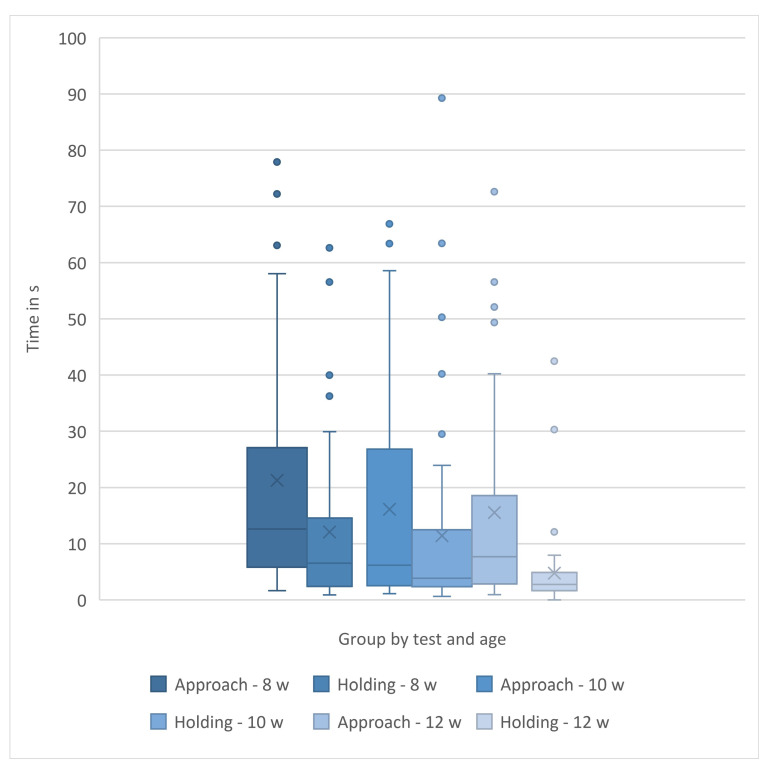
Approach latencies and holding times by week: The distribution of first approach latencies and longest holding times for kittens who approached and did not have extreme (>120 s) holding times (N = 39 for approach times; N = 46 for holding times). The box and whisker plots show the means (x), the medians (lines), the box contains 50% of the scores, and the values for 75% of index scores (whiskers) for each group. See text for SD/SE and statistical interpretation.

Finally, if any kitten with an extreme holding time at any week was removed from the data across all weeks, the longest holding attempt significantly differed at each of the three ages tested and *decreased* with age (N = 45) (ANOVA F = (2,88) = 5.00, *p* = 0.009) (see Table 2 and Figure 5B and Figure 6).

**Figure 7:**
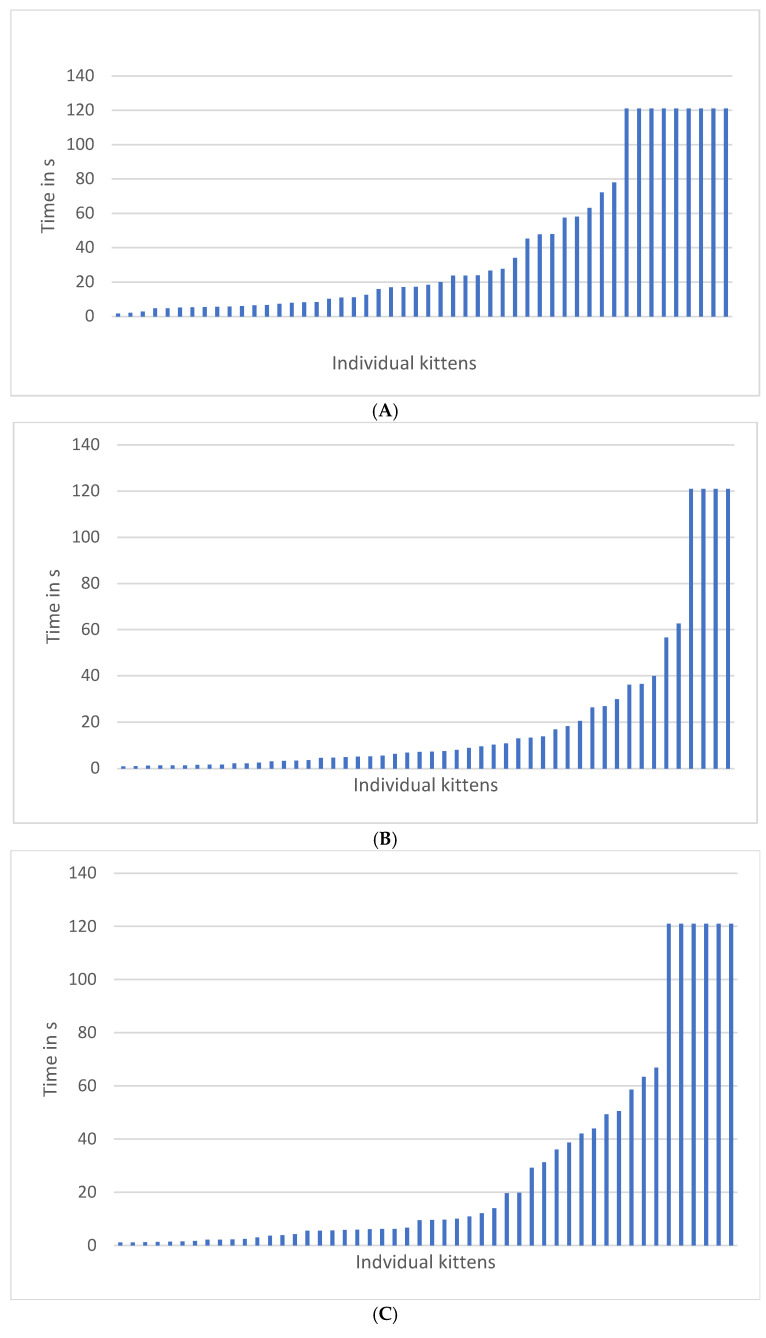

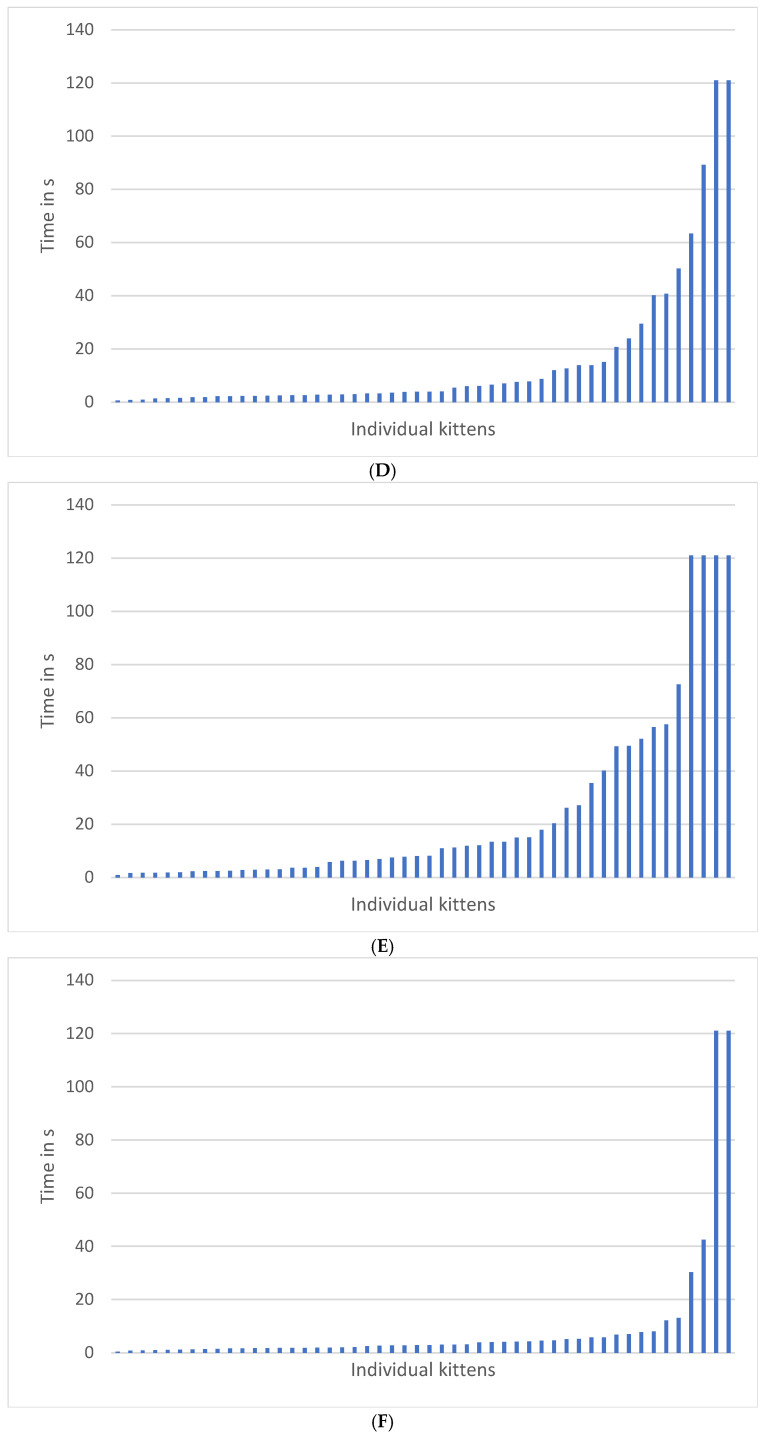
Distribution of (**A**) approach latencies at 8 weeks, (**B**) holding times at 8 weeks, (**C**) approach at 10 weeks, (**D**) holding at 10 weeks, (**E**) approach at 12 weeks, and (**F**) holding at 12 weeks. Each figure shows the data for all 50 kittens. Lines represent individual kittens not ordered by kitten ID or enrollment date. The number of kittens that did not approach decreased from 9 to 4 individuals in the approach test from 8 to 12 weeks. The number of kittens that did not stay in the lap crevice decreased from 4 to 1 individual in the holding test from 8 to 12 weeks. All kittens shown at the 120 s mark did not approach in the approach test or did not move from crevice of human lap in holding test.

Regardless of the way holding time data were analyzed, holding times *decreased* as the kittens aged from 8 to 12 weeks. The patterns of approach and holding times are graphed by testing week in Figure 7. All feather, approach, and holding data are summarized in Table 2.

With respect to whether the dam was present when the kittens came to the PEIHS (N = 27) or in the foster home (N = 21), there was an effect only on holding time at 8 and 10 weeks, but not at 12 weeks for either condition. Kittens were held longer if they had *not* had a dam with them at the PEIHS (8 weeks; Kruskal–Wallis, H = 7.12 (1, N = 46), *p* = 0.008; 10 weeks: Kruskal–Wallis, H = 9.63 (1, N = 48), *p* = 0.002) or in the foster home (8 weeks: Kruskal–Wallis, H = 11.80 (1, N = 46), *p* = 0.0006; 10 weeks: Kruskal–Wallis, H= 8.82 (1, N = 48), *p* = 0.003).

#### 3.3.3. Approach Latency as Related to Other Behavioural Tests

**Figure 8:**
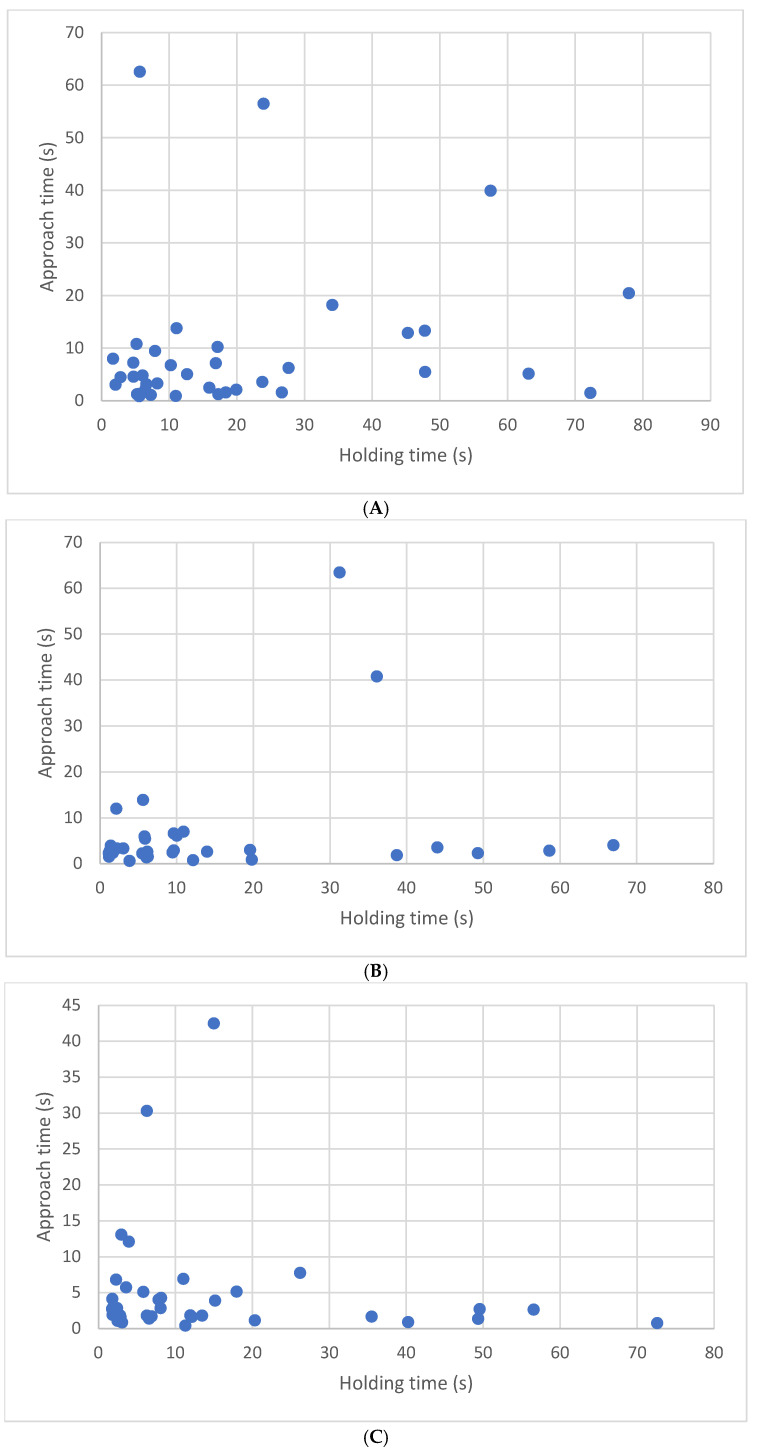
Approach latencies vs. holding times by week: Approach latencies and holding times (and respective regression equations) at (**A**) 8 weeks (ŷ = 0.13*X* + 7.03), (**B**) 10 weeks (ŷ = 0.20*X* + 4.21) and (**C**) 12 weeks (ŷ = −0.06*X* + 5.93) for kittens participating in the approach test.

Data for latency to approach and longest time tolerating holding can be found across weeks in Figure 8. To determine whether a predictive relationship existed between approach latency and holding duration, we examined the data of the 39 kittens that approached using Pearson correlations. There were no significant correlations between latency to approach a novel human and holding duration at 8 weeks (r(37) = 0.19, *p* = 0.27), 10 weeks (r(37) = 0.31, *p* = 0.24), or 12 weeks (r(37) = −0.12, *p* = 0.47). Latency to approach a novel human did not predict holding durations (8 weeks, r^2^ = 0.03 (Figure 8A), 10 weeks, r^2^ = 0.09 (Figure 8B), 12 weeks, r^2^ = 0.01 (Figure 8C).

### 3.4. Results of Physiological Assays

No significant correlation was found between HCC and RTL for study kittens (Pearson correlation; r(43) = 0.13, *p* = 0.39).

#### 3.4.1. HCC

The predictive value of HCC on the behavioural measures assessed in the interactive object test, approach to a novel human test, and the holding test were explored using correlation matrices and ANOVAs.

**Figure 9:**
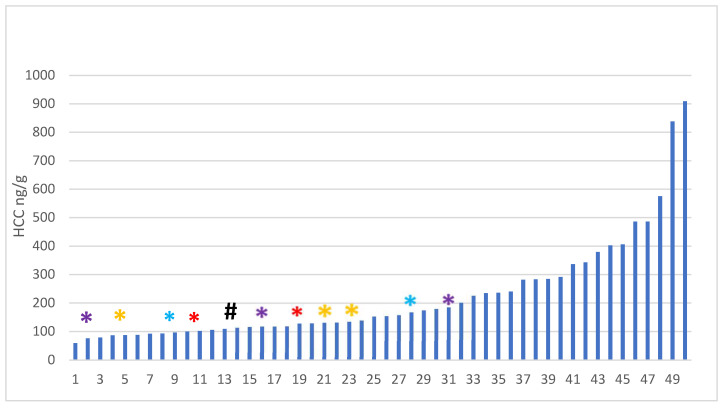
HCC by kittens: Plot of HCC by individual kitten plotted from low to high HCC. The starred HCC are the for the kittens who did not approach (4 of which also had holding times of >120 s). Sibling groups are noted by colour (*). The # indicates a kitten that did not approach, unlike its siblings.

When analyzing the data for the kittens who approached the novel human, there are no significant associations between HCC and latency to approach or duration of holding at any age. However, there was a considerable range of HCC across all kittens (Figure 9).

The group of kittens that did not approach a novel human was different not just in behaviour but also in terms of HCC. Kittens who did not approach novel humans had low HCC and tended—with one exception—to be part of sibling groups. In other words, if a kitten did not approach, at least one of their siblings also did not approach novel humans, with one exception (Figure 9).

**Figure 10:**
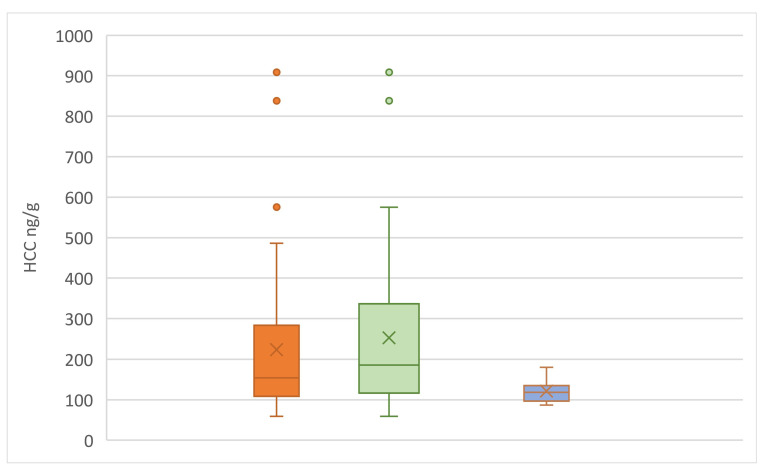
HCC across subgroups of kittens: Summary data for HCC across the entire group of kittens (orange), with kittens who did not approach removed (green), and for those of the kittens that did not approach on at least one occasion (plum). The box and whisker plots show the means (x), the medians (lines), the box contains 50% of the scores, and the values for 75% of index scores (whiskers) for each group. See text for SD and statistical interpretation.

In Figure 10, kitten HCC were compared between the various groups of kitten datasets—all the kittens, kittens without the non-approachers, and just the non-approachers. Kittens who did not approach at least once over the three test periods had a significantly lower mean HCC than the rest of the kittens, while the group of kittens where everyone approached had significantly higher HCC (Mann–Whitney U; *p* = 0.02; z-score = 2.32; U = 115).

There was no effect of sex on HCC.

**Figure 11:**
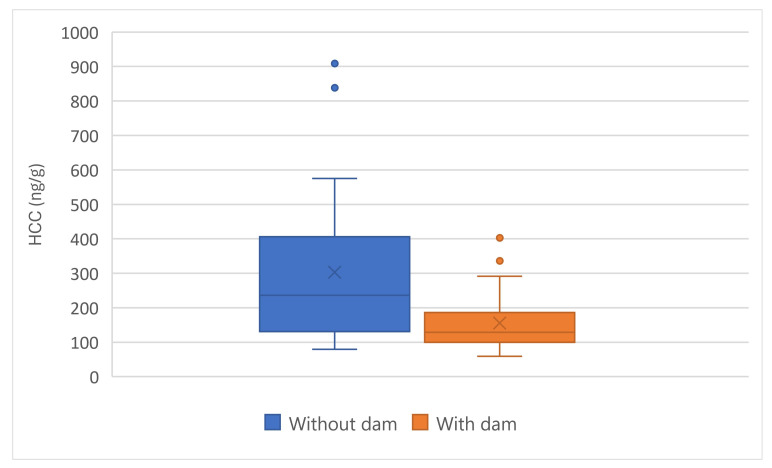
HCC by presence/absence of dam at HS: Effects of presence of dam upon arrival at PEIHS on HCC (N = 23 kittens without their dams and N = 27 kittens with their dams). The box and whisker plots show the means (x), the medians (lines), the box contains 50% of the scores, and the values for 75% of index scores (whiskers) for each group. See text for SD and statistical interpretation.

The presence of the dam at presentation to the PEIHS significantly affected HCC (F (1, 48) = 9.76, *p* = 0.003). Kittens raised with their dams had lower HCC than did those without their dams (Figure 11).

**Figure 12:**
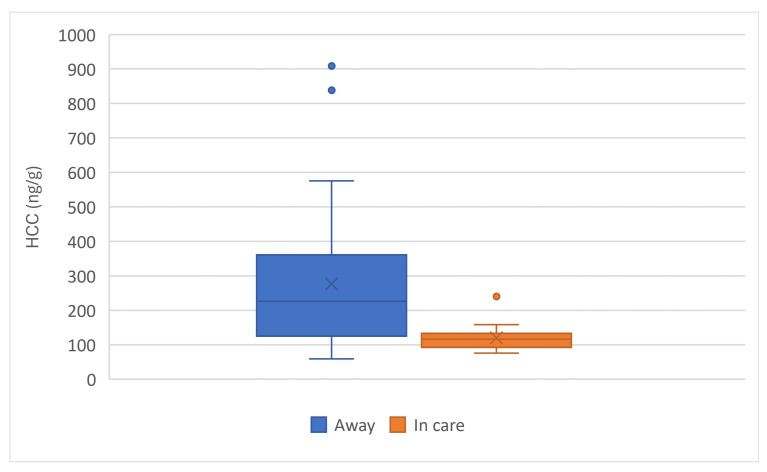
HCC by place of birth: Comparison of HCC by place of birth (N = 33 kittens born away and N = 17 kittens born in care). The box and whisker plots show the means (x), the medians (lines), the box contains 50% of the scores, and the values for 75% of index scores (whiskers) for each group. See text for SD and statistical interpretation.

When comparing HCC by place of birth, kittens born in care at the PEIHS (n = 17) had lower HCC than those born away (N = 33) (Figure 12). There was a significant effect of place of birth on HCC (F (1, 48) = 10.0, *p* = 0.003).

With respect to the number of days that kittens spent in foster care, prior to 8-week testing, a significant correlation was found between the number days spent in foster care (prior to testing) and HCC (r(48) = −0.32, *p* = 0.03), revealing that more time spent in foster care was associated with lower HCC.

HCC was compared with both approach latency to the feather toy and time playing with the feather toy for weeks 8, 10, and 12, for kittens who approached the toy. There were no significant Pearson correlation measures for approach latency and time spent playing for kittens who approached or not.

#### 3.4.2. RTL

The predictive value of RTL on the behavioural measures assessed in the interactive object test, approach to a novel human test, and the holding test were explored using correlation matrices. The associations between RTL and early-life factors such as whether the dam was with the kittens when they presented to the humane society, whether the kittens were surrenders or strays, et cetera, were assessed using separate ANOVAs. An additional 32 blood samples were collected to measure RTL for later-season kittens to test for effects of being born early or late in the season on RTL, but behaviours and early-life factors were assessed only for the original 50 kittens.

Correlations between RTL and total time engaged with the feather toy at 8, 10, and 12 weeks, were all weak, but significant when kittens who did not play with the toy (time = 0 s) were included (Pearson correlation: r (47) = −0.38 *p* = 0.007; r (47) = −0.31, *p* = 0.03; r (47) = −0.34, *p* = 0.017, respectively). One kitten lacked an RTL measurement. When the kittens with 0 s play times were removed, correlations between RTL and total time engaged with the feather toy at each of the three time periods were weak and significant for 8 and 10 weeks, only (Pearson correlation: r(29) = −0.47, *p* = 0.01; r(34) = −0.49, *p* = 0.003; r(40) = 0.27, *p* = 0.10, respectively). For no comparison was more than 20% of the variation explained.

**Figure 13:**
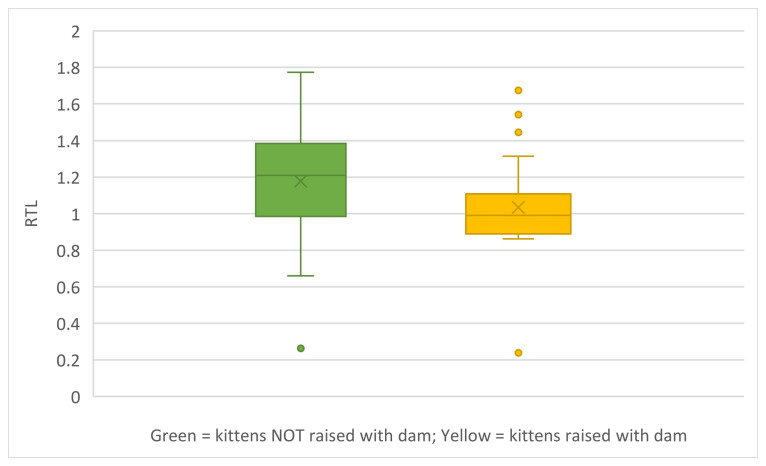
RTL by presence (N = 29)/absence (N = 21) of dam during presentation at HS: RTL was significantly lower for kittens with dams in their foster home. The box and whisker plots show the means (x), the medians (lines), the box contains 50% of the scores, and the values for 75% of index scores (whiskers) for each group. See text for SD and statistical interpretation. RTL for kittens that were surrendered were compared to those that were strays. There was no difference in RTL depending on intake status (Mann–Whitney U test; U = 294; z-score is −0.09; *p* = 0.93).

RTL was compared for kittens raised with their dams and kittens raised without their dam for the kittens tested behaviourally. Kittens raised with their dams had shorter RTLs than kittens raised without their dams (Mann–Whitney U-test; U = 183; z-score = 2.17; *p* = 0.03) (Figure 13).

With respect to the number of days that kittens spent in foster care, there was a significant but weak negative correlation between number of days in foster care and RTL (Pearson correlation; R = −0.21; *p* = 0.04).

There was no effect of sex on RTL for either the kittens tested behaviourally or when the full 81 samples (samples from kittens in the study and from the additional kittens sampled but not enrolled in behavioural testing) were considered (2 of the additional samples lacked sex information) (full dataset: Welch Two-Sample *t*-test; t = −0.54, df = 73.51, *p*-value = 0.59). There was no effect of timing during the season for sampling on telomere length (Welch Two-Sample *t*-test; t = 1.88, df = 63.43, *p*-value = 0.06).

Table 4 presents a summary of the significant findings. Table 5 presents a summary of all results.

## 4. Discussion

Effects of early-life stressors and deprivation are apparent in the homeless kittens on PEI. The amount of time spent in foster care, presence of dam in the foster home, interactions with dogs in the foster household, whether the kitten was a captured “stray” or a known “surrender”, and age at behavioural testing affected behavioural and/or physiological assays.

In general, there was decreased latency to play with the feather toy and more time spent in play with it with increasing age, as expected [35,97,98]. With respect to this interactive play, we did not find major behavioural differences between kittens separated from their dams and those kept with them except for the 10-week test, when kittens with dams approached the toy more quickly. Extensive research has been conducted on the topic of kitten play [35,97,99,100], and maternal engagement has been shown to facilitate interactive play with toys, but kittens separated from their mothers at ~5 weeks have shown an increase in object play compared with kittens separated later, post-weaning [34]. This increase in object play was considered a facultative response to early weaning, in the absence of other life stressors, which is concordant with the finding that early weaned kittens may hunt earlier [101]. Rodent studies have shown that maternally separated pups showed moderately enhanced sustained attention [18], which may translate into increased attentional focus on an interactive toy. In our study, many of the kittens may have been early weaned and others were at risk for lower quality or quantity of milk due to maternal stressors, but we lacked measures of these.

There were no other effects on latency to approach or time spent playing with the feather toy associated with the presence or absence of mothers. Early weaning has been associated with increases in social and object play [34,36], an effect we did not observe here for kittens without dams. Kittens that did not approach the feather toy were over-represented in the group of kittens that did not approach humans. Social play begins to develop in kittens at 3–4 weeks, and object play is fully honed by 7–8 weeks, with earlier object play accompanying early weaning [97]. Our combined results suggest that both interactive object play and social interaction may have been impaired in these kittens. This result may suggest compromised maternal nutrition during gestation and post-partum [33].

Interestingly, there was no effect of the presence of another cat with whom the kitten interacted in foster on kitten play with the feather toy or on latency to approach a stranger, but there was an effect of the presence of an interacting dog on each of these. Kittens that did *not* have an interacting dog played longer with the feather toy and approached humans faster. This finding may indicate that in this population of kittens, other interactive companion animals do not facilitate object play or interactions with people, and instead may redirect focus from these activities, possibly similarly to the result discussed above for rodents [18].

While we used methodology roughly adapted from Karsh [41] and Karsh and Turner [3], we had very different results. Contrary to our hypothesis, the PEIHS kittens experienced a decreased in approach latency at 10 or 12 weeks when compared with at 8 weeks, without a commensurate decrease between 10 and 12 weeks, and a *decrease* in holding times with age (even with lap-burrowing kittens removed), patterns that contrast from that found by Karsh and Turner for kittens handled across these time periods evaluated starting at 14 weeks. Our data may suggest that, for the PEIHS kittens, there was insufficient early exposure and/or other liabilities that interfered with the development of a shorter approach time and an increased holding duration with age.

We found an effect of having the dam present at intake at the PEIHS and in the foster home only in terms of holding time at 8 and 10 weeks. At these times, kittens who *lacked* a dam either at the PEIHS or at the foster home tolerated holding longer than did kittens with access to dams. Holding time still decreased across time, and the effect of absence of the dam was gone by 12 weeks. Many of our kittens may not have been handled prior to arriving in fosters, which may have affected the pattern of handling duration. It is possible that 8- and 12-week-old kittens who lacked dams had more or different early handling by humans and so stayed with them marginally longer at first. However, by 12 weeks, this effect was not sufficient to overcome whatever was driving the pattern where kittens allowed themselves to be held for decreasing amounts of time when tested at 8, 10, and 12 weeks.

We found no significant correlations between latency to approach a novel human and holding duration at 8, 10, or 12 weeks, as represented in Figure 8A–C. Our divergence in findings between latency to approach and holding times may further suggest that homeless kittens may be impaired in their ability to interact intimately and remain with humans, as they age, in a way that is not seen in kittens who lacked deprivation. If this finding is true, it is important. The differences between our findings and those of Karsh and Turner may be the result of subtle differences in the methodology. In their studies [3,41], kittens were handled daily for 15 or 40 min, depending on the study. During that time, they were returned to the person’s lap each time they decided to leave. Unlike the kittens in those studies, the PEIHS kittens had no formal handling protocol prior to testing. Additionally, the experimenters holding the kittens in the Karsh [41] and Karsh and Turner [3] studies were already known to the kittens, whereas the kittens in this study had not previously encountered the experimenter (with the possible exception of the brief exposures during the testing periods). That said, these differences in methodology are unlikely to be sole cause for the differences in performances between the two populations of kittens (well-cared-for laboratory cats vs. homeless rescue kittens with uncertain starts in life). It is more likely that epigenetic factors and differences in early-life exposure altered the way the rescue kittens responded to the tests.

The findings from Karsh and Turner [3] are in the direction that one would predict—that with age and exposure, latencies to approach humans should decrease, and their willingness to be held by a human should increase. In our kittens, latencies to approach decreased between 8 weeks and 10 and 12 weeks, without a further decrease between 10 weeks and 12 weeks. Yet, holding times *also decreased* for each time period examined, *including* at our last assessment at 12 weeks.

Our data are far more in line with Karsh and Turner’s data for holding time for their ‘genetically shy’ cats than the spectrum we expected. Approach and holding times did not correlate with or predict the other for our kittens. It is possible that laboratory kittens, born into a laboratory care environment, cared for daily, had at least passive daily exposure to humans, and with mothers fed a diet that meets the minimum gestational standards differ greatly from homeless kittens. Additionally, all the mothers of the laboratory cats in Karsh’s studies, were, themselves, laboratory cats, and so both mothers and kittens received basic nutrition and care throughout their lives without being subjected to any of the deprived conditions that could contribute to epigenetic effects that the mothers of the PEI homeless kittens were likely to experience.

Numerous studies [102,103,104,105] have shown that the extent to which kittens are ‘bold’, ‘friendly’, ‘outgoing’, et cetera, is determined by the father. McCune [102] found that cats fathered by bold, outgoing, friendly cats were more likely to explore inanimate objects and unfamiliar humans at 1 year and that such effects were enhanced by early handling. Given that all of the kittens in this study were homeless and that PEI has historically had a large population of unowned/homeless cats, it is possible that the breeding males in this population and other such populations disproportionately comprise cats who are not bold, friendly, or outgoing since human investment in such cats as pets may be minimal. If so, the temperament of the population of fathers of homeless kittens may be quite different than that for the fathers of ‘home- or purpose-bred’ kittens.

In the present study, the effects of maternal separation on the behaviour, telomere length (RTL), and hair cortisol concentrations (HCC) in kittens were investigated.

Higher HCC, which is often viewed as maladaptive or a marker for stress responses, was, as predicted, associated with less time spent in foster care, intake as a stray, and absence of the dam in foster care. Lower HCC, which is often viewed as preferred or adaptive, was associated with more time in foster care, intake as a surrender, and the presence of the mother in foster care. Yet, the kittens that did not approach or interact with novel humans had lower levels of a marker of cumulative stress response, HCC. Given that these behavioural data closely resembled those of Karsh and Turner [3] for ‘shy’ kittens (Karsh and Turner did not measure biomarkers), we need to question whether lower HCC here is neither adaptive nor desired, but instead is the result of a chronically blunted response to stressors and not simply behavioural ‘shyness’. Combined with the finding that HCC is associated with whether a kitten approached a novel human, higher HCC may be driving heightened ‘arousal’ or increased activity, possibly as an anxious, uncertain response that allows the kittens to attend to the surrounding conditions. If so, this outcome could be an adaptive response to an uncertain early environment. Such kittens may be labelled as ‘bold’, but the function of such behaviour may be to assess an uncertain environment rather than to directly interact. We lack ongoing data to test this hypothesis, but these correlations all cluster around factors affecting risk (i.e., less time spent in foster care, intake as a stray, and absence of their mother in foster care).

Low cortisol levels have received attention with respect to SHRP. SHRP has been extensively studied in other species with demanding maternal care, but there have been no studies on SHRP in kittens. Jensen et al. [106] noted that 2–4-week-old kittens exhibit physiological and behavioural plasticity with respect to temperature regulation to environmental temperature changes, but this effect is gone by 4 weeks. These data may suggest that the SHRP in kittens—if one exists—is over by 4 weeks, as is the case for dogs [52,107], for whom intensive maternal care extends the SHRP only to 5 weeks [52].

The increased cortisol levels observed in maternally separated kittens in this study likely stems from the heightened stress response during this critical period. However, for the subsample of the population with low cortisol (who are not outgoing), cortisol may be blunted due to chronic glucocorticoid elevation associated with chronic stressor exposure [27,58,59,60]. HCC is a summary measure of cortisol over 3 months, and in this study, HCC was measured at 8 weeks. Accordingly, HCC assayed glucocorticoids from midway through the 63-day gestation period and then through the first 2 months of life. If sufficiently high, the maternal stress experienced by this subpopulation of kittens, which was clustered in this study by sibling groups, could have blunted the response of the kittens to stress as a larger response to chronic stressors, and aberrantly affected the development of the general HPA response. This blunted pattern of response has been reported for human infants that were small for their gestational age [108]. Weight and gestational age data were not collected here.

These wide-ranging findings cloud any consistent argument for an SHRP in kittens in this dataset.

While there is no established direct mechanistic link between cortisol (or HCC) and RTL [109,110], we hypothesized that elevated HCC levels would be associated with decreased RTL concordant with findings in other studies [111,112,113]. However, telomere length is influenced by multiple factors, including genetics [114], oxidative stress [115], age [116], and nutrition [117], not just cortisol [109,110]. We found no association between HCC and RTL in these homeless kittens.

There are data suggesting that RTL can be elongated in childhood adversity [118]. In this study, kittens experiencing what we thought would be the most adverse environments—more time as a stray and less time in foster care; absence of their mother—had *longer* RTL, in contrast to our hypothesis. Whether our short time period for evaluation of such effects was sufficient or comparable to the human data is questionable.

In contrast to our contradictory finding that RTL is lower when the dam is present, another study on homeless kittens, where the kittens were in constant care, found no association between length of telomeres at 1 week or 2 months of age and orphan status (with dam or not), or the interaction of the two [88]. Telomere lengths do decrease with age across species [87,116], but the time spans measured should be meaningful in terms of lifespans, which may not have been the case in our study. It is possible that both we and Delgado et al. [88] simply did not measure telomeres over an age range likely to provide meaningful data for kittens.

As noted, our only significant associations with RTL were with play time with the feather toy at 8 and 12 weeks, with presence of the dam during foster, and with time spent in foster care. We found a weak, negative effect of time in foster care on RTL. Kittens with longer telomeres spent less time playing with the toy and, unexpectedly, did not have dams present. It is possible that in the PEIHS kittens, these findings hint at truncated play periods that have been reported to be associated with early fighting and/or independent hunting behaviours [101], especially for kittens who likely have pre- and perinatal compromised nutrition. If so, the association with absence of dams and less feather toy play in cats with longer telomeres may be an adaptive response. We lack data to test this hypothesis.

We did not find a significant effect of season or age on RTL when we compared our late season sampled kittens to those that underwent behavioural testing. We had fewer later season samples and a relatively small overall sample size. The analysis suggests that a larger study should confirm whether there is an effect of season—which may affect the nutrient and shelter environments—when the telomeres are measured across the kitten season.

Finally, the reason we collected data on RTL was not simply to test for early effects. Most measured effects of telomere length are on aspects of behaviour and longevity later in life (e.g., adults). Our original study design included long-term follow-up of the adopted kittens with quarterly assessments of potentially problematic behaviours. A change in how the kittens were handled for adoption at the PEIHS prohibited this, and the overwhelming majority of kittens were lost to follow-up. It is also possible, given the results Delgado et al. [88], that RTL is not a useful measure in young kittens, or that RTL is the result of complex interactions that cannot be adequately assayed in small populations.

## 5. Limitations

There were a number of limitations to this study, and our data should be regarded in this light.

1. All kittens in this study were homeless and from PEI, a small, rural, economically challenged Canadian province that can have extreme winters. This population may not be representative of any other population of kittens, even other homeless kittens who may be raised in a less harsh winter environment and, therefore, experience a different resource environment.
2. Because of the requirement to enrol sufficient kittens over the course of the study and to test each kitten three times over 5 weeks, we were limited in the time frame for this study. The kittens we tested behaviourally were all born in early-to-mid-kitten season on PEI. It is possible that the kittens born at the end of the reproductive season differed behaviourally from these kittens; however, there was no difference in telomere length between kittens sampled early in the season and later in the season.
3. We modelled our testing methodology for having kittens approach and be held by humans after Karsh’s methodology since it was some of the only methodology available. In adapting this methodology to a ‘field’ study from a laboratory study, numerous changes were made. For example, the laboratory kittens changed rooms but did not get into a car and leave the lab to reach the testing facility. Lab kittens also likely had more consistent care, handling, and exposure to humans and environments across their lives than ours did. However, these are the only close-to-comparable published data available to discuss, and they differed greatly. Our methodology is reported in detail and should be replicable by anyone in any environment. No special equipment is needed for any group of cats—owned, laboratory, or rescue—to be tested in a companion study.
4. We discuss the effects of how the kittens came to the PEIHS, number of days in foster care, presence or absence of the dam during foster, et cetera. However, shelters are dynamic places—no two cats have the same experience. Fosters, by definition, do not provide each animal in their care with an identical experience to another animal in foster, and we had no way to ask or evaluate the extent to which foster-carers held and petted kittens each day or whether this varied with age and individual kitten. All of the foster-carers in this study had a long-time association with the PEIHS, were experienced, and were devoted to the mission of the PEIHS and to this study. Beyond knowing this, we had no way to evaluate the quality of the behavioural or physical environment provided by any foster. In a small study such as this one, these are valid concerns.
5. We could not evaluate the nutritional status of these cats. Nutritional status is one factor that may differ between stray vs. surrendered kittens, and the categories we used here act as surrogates for a number of potentially important mechanisms affecting behaviour. RTL has also been affected by nutritional status [117,118].
6. We did not weigh these kittens at each visit. Retrospectively, this was an error since it may have given us insight into whether HCC and RTL were associated with any ‘cost’ to growing, and whether nutritional status was a potential concern.
7. Finally, we had anticipated being able to follow these kittens throughout at least the first year and preferably the second year of life at 3-month intervals. The manner in which the kittens were adopted differed from our original plan and rendered our planned quarterly follow-up, and meaningful later assessment of early RTL, impossible.

## 6. Conclusions

Our study suggests that there is an intricate interplay between maternal separation, early-life stressors, hormonal stress responses, telomere length, and behavioural responses to humans and interactive play in kittens.

Considered as a group, these results suggest that developmental changes in play behaviour may be indicative of underlying epigenetic mechanisms influenced by early-life stress. There are few studies (this one; [3,66,88]) that have examined the effects of early-life stressors on any aspect of quantifiable behaviours as cats move through kittenhood. In an extensive survey study, Ahola et al. [66] found that home-bred kittens kept until 15 weeks in their natal home have decreased owner-reported aggressiveness and oral obsessive–compulsive behaviours as they matured compared to kittens adopted between 8 and 12 weeks. It is unlikely that laboratory cats, pet cats, and homeless cats experience the same early-life stressors, that they are all from the same genetic and/or epigenetic population, or that they respond the same to these stressors. Additionally, our kittens came from the first group of kittens taken in by the PEIHS in an organized way after 2 years of COVID-19 restrictions. It is possible that this period amplified stressors and compounded their effects on epigenetics in ways we cannot know.

Our study flags an urgent need to experimentally investigate early-life stressor effects across kittens from a range of rearing conditions in a manner that allows these effects to be evaluated with respect to the adult behaviour of the cat. We evaluated only two types of interactions with unfamiliar humans: willingness to approach, which is the first step in exploring and actual interacting, and willingness to be gently and safely held, an essential step in providing care and love to cats. The results in neither test were as expected. Our data on willingness to be held by novel human are not even in the direction we anticipated. We expected that kittens would approach more quickly as they matured (which they did, but only between 8 and 10 weeks; time to approach was stable between 10 and 12 weeks) and allow themselves to be held longer with age and exposure (which they did not). These data suggest that homeless kittens may be particularly at risk for problematic exploratory and interactive behaviours involving humans, whether or not the kittens perceive these behaviours as adaptive. These factors could affect the adoptability and retention in households. Such issues should be urgently explored, given the number of homeless and feral cats in the worldwide cat population. Understanding these dynamics may have broader implications for our understanding of early-life stress and its effect on developmental outcomes in various species, including humans.

## Figures and Tables

**Table 1:** Summary results from the literature on early kitten behaviour.

Authors	Type of Study	Feline Subjects	Intervention(s)	Measures Assessed	Results
Seitz 1959 (as reviewd in [29])	Cohort	N = 18 kittens of homeless cats that were brought to laboratory ~2 weeks before delivery; 6 mother cats, each with 3 kittens, 1 kitten from each litter placed into different treatment groups; kittens tested when reached adulthood at 9 months	Kittens removed from mothers at three different ages: 2 weeks, 6 weeks, and 12 weeks and then subsequently placed into individual cages	Observed behaviours during daily exercise periods with 2 other kittens present, emergence from their home cages, jumping from an unfamiliar ledge, behaviours secondary to a variety of unfamiliar enclosures, responses to intense light and sound stimuli, and feeding behaviours	Performance of male and female cats indistinguishable; 6-week-old kittens most reluctant to leave home cages (followed by 12-week-old and then 2-week-old kittens); 2-week-old kittens generally showed the most random movement and least goal-directed movement and reacted to novel stimuli with greater anxiety, and they also had increased frustration, increased aggression, slower learning, decreased sociability, and decreased adaptability; 6-week-old kittens generally most reluctant to explore new environments
Karsh et al., 1983, 1984, 1988 [3,40,41]	Longitudinal	Various sized groups of laboratory kittens handled for 15 or 40 min per day from 3 to 14 weeks, 7–14 weeks, and no handling Other groups were handled from 1 to 5 weeks (N = 18), 2–6 weeks (N = 21), 3–7 weeks (N = 19), and 4–8 weeks (N = 17) and consisted of timid and bold cats that were evaluated for holding time by a novel person. A third group comprising laboratory bred kittens that were home reared from 4 weeks and handled 1–2 h/d (4–8 x longer than laboratory kittens). These kittens were brought into the lab at 14 weeks for approach and holding testing.	Kittens in all groups asked to approach novel human and be held by them under laboratory protocols	Approach time (seconds (s)) to novel humans and holding time (s) without struggle in kittens at 14 weeks.	Kittens handled 3–14 weeks vs. 7–14 weeks approached more quickly (11 vs. 41 s) and were held longer (41 vs. 24 s), and both did better than kittens not held at all for first 14 weeks. Effects for both approach time (shorter) and handling time (longer) were enhanced at 14 weeks if time of handling was increased to 40 min per day, at which point the 3–14 week and 7–14-week groups were indistinguishable. Kittens handled in overlapping intervals from weeks 1–5 though 4–8 were held the longest if exposed at weeks 2–6 and 3–7 (which were not different from each other). Holding times for 1–5 and 4–8 week kittens were not different but were lower than for weeks 2–6 and 3–7. Holding time followed the same pattern but was enhanced for all 4 periods if the kittens were bold; for shy kittens the response was flat. At 14 weeks, kittens from home environments approached researchers immediately, crawled into their laps and fell asleep, something no laboratory kitten did.
Lowe and Bradshaw, 2002 [46]	Longitudinal	29 random-bred, household cats from 9 litters tested at 2, 4, 12, 24, and 33 months by being held by a standard, unfamiliar person	Holding test adapted from Karsh, 1984; cat placed in stranger’s lap and head and back stroked for 60 s or until cat struggles; repeated within the 60 s window if leaves.	Handling time and number of escapes noted. The amount of handling the kitten had between 1 and 2 months and then ranking of kittens in litter based on early handling assessed via questionnaire from breeder.	Median handling time in 2nd month = 1.5 h (0.33–2.5 h) per day. No kittens showed signs of distress at 2 months. At 2 months, cats handled the least in the second month of life had the most escape attempts. By 4 months, cats handled the least had the fewest escape attempts. Escape attempts at 4 months were correlated with those at 24 and 33 months.
Marchei et al., 2009 [44]	Longitudinal	Oriental/Siamese/Abyssinian breed kittens (N = 43) compared with Norwegian forest cat kittens (N = 39) tested from 4th through 10th weeks of age in an open field test	Tests conducted in owner of queen’s cattery; 12 min focal animal sampling video recorded.	Heart rate and temperature measured before and after test; behaviours during test measured using focal animal sampling.	Norwegian forest cats had earlier thermoregulatory capabilities. These kittens spent more time exploring the open field and tried to escape more. Higher locomotion scores and longer time spent standing was noted in Oriental group. Oriental group kittens also had higher heart rates in response to an open field challenge and declined faster with respect to exploration and locomotion.
Marchei et al., 2011 [45]	Longitudinal	Oriental/Siamese/Abyssinian breed kittens (N = 43) compared with Norwegian forest cat kittens (N = 39) tested from 4th through 10th weeks of age in an open field test	Tests conducted in owner of queen’s cattery; 12 min focal animal sampling video recorded. At 6 min, a loaded spring (meant to be a threatening object) was released and kittens’ behaviours were recorded for 6 additional minutes.	Heart rate and temperature measured before and after test; behaviours during test measured using focal animal sampling.	Norwegian forest cat kittens had an active-coping strategy where they avoided scary situations. This strategy correlated with their high scores for exploration and escape attempts. Oriental kittens responded to the stimulus by decreased locomotion (passive coping strategy), but also with tachycardia. They may not have become behaviourally aroused to the same level as did the Norwegian forest cats but were still concerned, and maybe more concerned as indicated by physiological arousal.
Martinez-Byer et al., 2023 [43]	Case–control	N = 62 homeless rescue kittens from 19 litters; kittens tested between 9 and 10 weeks of age	Kittens were either mother-reared (N = 32), hand-raised orphans with siblings (N = 14), or hand-raised singletons (N = 16)	Three behavioural tests—the struggle test (how long the kitten tolerated being held out at arm’s length), the meat test (defensive behaviours in protection of a piece of meat), and the separation/confinement test (placed into travel carrier for 5 min, thermal imaging performed before and after test)	Males larger than females at time of testing, no morphologic or physiologic differences noted between treatment groups; orphaned kittens (both with and without siblings) struggled sooner and showed increased vocalization and motor activity during the separation/confinement test; no notable differences between orphans with and without siblings
Graham et al., 2024 [47]	Cross sectional	N = 235 foster kittens aged 7–9 weeks; N = 72 foster parents	Foster parents completed online survey	Surveys involved questions about the foster parents such as household demographics, previous fostering and animal experience, their personalities, litter details, and individual kitten signalments, behaviours, and experiences	Kittens that were fearful upon intake were more likely to be fearful of unfamiliar people and objects at adoption age

**Table 2:** Age-related changes in the feather test, approach to novel human test and the holding test. The rows in italics represent analyses where we removed only the week(s) with the extreme (>120 s) holding times from the analysis (top row) and where we removed any cat from the holding time dataset that had an extreme holding time at any test interval (bottom row). Values represent mean (±standard deviation (SD)[SE]). Times are in s. Superscripts represent comparisons that differ significantly (*p* </= 0.05). Within a row, means without a common superscript differ statistically. See text for details and analysis.

	8 Weeks	10 Weeks	12 Weeks
**Interactive Object/Feather**			
Latency (s) to engage with feather toy only for kittens who approached it across all ages (N = 27)	15.73 (±17.74[3.41]) ^a^	8.43 (±14.51[2.79]) ^b^	5.16 (±8.12[1.56]) ^c^
Time to engage with feather toy only for kittens who approached it across all ages (N = 27)	35.02 (±18.39[3.53]) ^a^	49.54 (±16.65[3.2]) ^b^	53.03 (±11.23[2.16]) ^c^
Latency (s) to engage with feather toy only for kittens who approached both the toy and humans (N = 26)	16.05 (±18.01[3.53]) ^a^	8.63 (±14.76 [2.89]) ^b^	5.424(±8.27[1.62]) ^c^
Time (s) engaged with feather toy only for kittens who approached both the toy and human (N = 26)	35.58 (±18.52[3.7]) ^a^	49.30 (±16.93[3.38]) ^b^	52.88 (±11.42[2.28]) ^c^
**Approach**			
Latency (s) to approach a novel human (see Figure 5A and text) (N = 39)	20.3 (±20.4[3.29]) ^a^	14.9 (±18.8[3.01]) ^b^	14.8(±17.4[2.79]) ^b^
**Holding**			
Maximum duration (s) held across 3 attempts; all kittens included (N = 50)	20.8 (±32.9[4.65]) ^a^	15.8 (±27.8[3.93]) ^a^	9.5 (±24.1[3.41]) ^b^
Maximum duration (s) held across 3 attempts with kittens that did not approach removed across all ages (N = 39)	9.6 (±13.9[2.23]) ^a^	10.1 (±21.8[3.49]) ^b^	5.04 (±8.0[1.28]) ^c^
Maximum duration (s) held across 3 attempts for only the kittens that did not approach for at least 1 age assessed (N = 11); 4 of these kittens had holding times excessive (> 120 s) holding times for at least 1 age	60.4 (±48.7[18.3]) ^a^	35.9 (±37.6[11.39) ^b^	25.3 (±47.3[14.33]) ^c^
Maximum duration (s) held across 3 attempts; kittens removed with holding times > 120 s in any test period (N = 45)	12.14 (±14.7[1.81]) ^a^	10.33 (±17.3[1.54]) ^b^	5.04 (±7.4[1.1]) ^c^

**Table 3:** Status of dam and latency to approach toy and time in s engaged with feather toy during a 1 min interactive test. Values represent mean (±SD). The significant group difference is denoted with an asterisk. See text for details and analysis.

**Latency to Approach (s) the Feather Toy by Age and Presence of Dam Through 8 Weeks of Age**		
	**Dam Absent (n =16)**	**Dam Present (n = 11)**
8 weeks	16.90 (±18.35)	14.03 (±17.55)
10 weeks	13.10 (±17.37) *	1.63 (±3.01) *
12 weeks	7.20 (±9.80)	2.17 (±3.34)
**Time (s) Engaged with the Feather Toy by Age and Presence of Dam Through 8 Weeks of Age**		
	**Dam Absent (n =16)**	**Dam Present (n = 11)**
8 weeks	29.38 (±17.73)	43.23 (±16.83)
10 weeks	44.55 (±19.97)	56.81 (±4.98)
12 weeks	49.21 (±13.78)	57.85 (±4.21)

**Table 4:** Summary comparison of significant associations for behavioural and demographic findings for HCC and RTL.

Factor	HCC	RTL
HCC × RTL	NS	NS
Open field interactive feather test	NS	**Significant *but weak* correlations** at 8 and 10 weeks: shorter RTL, longer play time. **Significant *but weak* correlations** at 8, 10, and 12 weeks: shorter RTL, longer approach time.
Approach test latency	**Significant:** lower HCC: non-approachers [higher HCC: approachers]	NS
Presence of dam	**Significant:** lower HCC: presence of dam [higher HCC: absence of dam]	**Significant:** shorter RTL: presence of dam [longer RTL: absence of dam]
Intake status	**Significant:** lower HCC: surrender [higher HCC: strays]	NS
# days spent in foster care prior to testing	**Significant:** lower HCC: more days spent in foster care [higher HCC: fewer days spent in foster care]	**Significant but weak:** shorter RTL: longer time in foster care [longer RTL: less time in foster care]

**Table 5:** Combined summary finding for all analyses reported. No effects in italics.

Interactive Object/Feather Toy	
**Measure**	**Review of Significant Outcomes**
Latency to approach feather toy	Significantly shorter approach latency at 12 weeks than at 8 and 10 weeks
Time spent in engaging with feather toy	Time spent engaging with the feather toy significantly increased with each age group
Time spent engaging with feather toy x presence of dam	Latency to approach feather toy was shorter at 10 weeks for kittens with dams
**Measure**	**Review of significant outcomes**
Time (latency) to approach an unfamiliar human	10- and 12-week-old kittens approached significantly more quickly than did 8-week-old kittens.
Holding time	Longest holding attempt differed significantly at each age and *decreased* with each age
Association between latency to approach novel human and holding duration.	*No correlation between latency to approach and holding time*
**HCC and RTL**	
**Measure**	**Review of significant outcomes**
Association between HCC and RTL	*No correlation between HCC and RTL*
Effects of HCC on latency to approach a novel human	*No effect of HCC on latency to approach at any age*
Effects of HCC on holding time	*No effect of HCC on holding time at any age*
Effects of presence of dam on HCC	Presence of dam had lower HCC
Effects of intake style (surrendered vs. stray) and HCC	Lower HCC in surrendered kittens; higher HCC in strays
Effects of place of birth (away vs. in care) and HCC	Lower HCC in kittens born in care; higher HCC in kittens born away
Time spent in foster care and HCC	More time in foster care associated with lower HCC
RTL and time spent with feather toy	Shorter RTL, more time engaged with feather toy (longer RTL, less time engaged with the feather toy) at 8 and 12 weeks, but no more than 20% of the variance was explained for any significant correlation.
Effects of presence of dam on RTL	Shorter RTL associated with presence of dam
Effects of time spent in foster care on RTL	Shorter RTL weakly associated with longer time in foster care
Effects of intake style (surrendered vs. stray) and RTL	*No effects with respect to intake status*

## Data Availability

Data are available from the corresponding authors upon request.
